# Advancing Studies on Neighborhoods and Brain Health: New Methodologies and Underrepresented Groups

**DOI:** 10.1093/geroni/igaf122.945

**Published:** 2025-12-31

**Authors:** Lilah Besser, Jessica Finlay, Jana Hirsch

## Abstract

Evidence is building for associations between neighborhood social/built environments and brain health in older adults. Studies show that individuals in more deprived neighborhoods have faster cognitive decline and increased dementia risk, while those in neighborhoods with more health-promoting resources (e.g., parks, destinations) have slower cognitive decline and lower dementia risk. However, prior studies had some methodological limitations by not considering critical periods of exposure, underrepresented groups, biological measures of brain health, neighborhood environments as structural determinants of health, or neighborhood environments as effect modifiers. Additionally, the field would benefit from incorporation of technologies such as GPS tracking, which characterize how frequently and in what manner individuals are exposed to community environments. This symposium presents five studies that address these scientific gaps. Dr. Magid will present on five domains of spatial social polarization (SSP) and the projected change in dementia incidence should SSP be eliminated. Dr. Moored will present on the differential findings for an intervention to improve memory among older adults (Experience Corps) based on their neighborhood resources. Dr. Hyun will present on associations between GPS-derived activity space measures of community environment exposure and cognitive function in the Einstein Aging Study. Dr. Sadler will present on the development of structural built environment measures across the life course, which will be linked to cognitive outcomes in the Healthy Aging in Neighborhoods of Diversity across the Life Span cohort. Lastly, Dr. Besser will present on associations between neighborhood greenness in midlife and late-life brain imaging measures in the Multi-Ethnic Study of Atherosclerosis.

